# Advanced glycation end products and cognitive impairment in schizophrenia

**DOI:** 10.1371/journal.pone.0251283

**Published:** 2021-05-26

**Authors:** Akiko Kobori, Mitsuhiro Miyashita, Yasuhiro Miyano, Kazuhiro Suzuki, Kazuya Toriumi, Kazuhiro Niizato, Kenichi Oshima, Atsushi Imai, Yukihiro Nagase, Akane Yoshikawa, Yasue Horiuchi, Syudo Yamasaki, Atsushi Nishida, Satoshi Usami, Shunya Takizawa, Masanari Itokawa, Heii Arai, Makoto Arai

**Affiliations:** 1 Department of Psychiatry and Behavioral Sciences, Schizophrenia Research Project, Tokyo Metropolitan Institute of Medical Science, Setagaya-ku, Tokyo, Japan; 2 Department of Psychiatry and Behavioral Science, Juntendo University Graduate School of Medicine, Bunkyo-ku, Tokyo, Japan; 3 Department of Psychiatry, Tokyo Metropolitan Matsuzawa Hospital, Setagaya-ku, Tokyo, Japan; 4 Department of Psychiatry, Takatsuki Hospital, Hachioji, Tokyo, Japan; 5 Research Center for Social Science & Medicine, Tokyo Metropolitan Institute of Medical Science, Setagaya-ku, Tokyo, Japan; 6 Graduate School of Education, University of Tokyo, Bunkyo-ku, Tokyo, Japan; 7 Division of Neurology, Department of Internal Medicine, Tokai University School of Medicine, Isehara, Kanagawa, Japan; Chiba Daigaku, JAPAN

## Abstract

Advanced glycation end products play a key role in the pathophysiology of schizophrenia. Cognitive impairment is one of the central features of schizophrenia; however, the association between advanced glycation end products and cognitive impairment remains unknown. This study investigated whether advanced glycation end products affect the cognitive domain in patients with schizophrenia. A total of 58 patients with chronic schizophrenia were included in this cross-sectional study. Plasma advanced glycation end products were measured using high-performance liquid chromatography (HPLC). Neuropsychological and cognitive functions were assessed using the Wechsler Adult Intelligence Scale, Third Version, and the Wisconsin Card Sorting Test Keio-FS version. Multiple regression analysis adjusted for age, sex, body mass index, educational years, daily dose of antipsychotics, and psychotic symptoms revealed that processing speed was significantly associated with plasma pentosidine, a representative advanced glycation end product (standardized β = -0.425; *p* = 0.009). Processing speed is the cognitive domain affected by advanced glycation end products. Considering preceding evidence that impaired processing speed is related to poor functional outcome, interventions targeted at reducing advanced glycation end products may contribute to promoting recovery of patients with schizophrenia as well as cognitive function improvement.

## Introduction

Advanced glycation end products (AGEs), generated via non-enzymatic glycation of reducing sugars with proteins or lipids, have been implicated in various diseases, including cardiovascular events in diabetes mellitus [1], chronic renal failure [2], and Alzheimer’s disease [3]. Previous cross-sectional studies revealed that plasma pentosidine, a representative AGE, is associated with schizophrenia [4, 5] and is a useful biological marker for the treatment-resistant-like phenotype [6]. Furthermore, a clinical trial using pyridoxamine, which is one of the three forms of vitamin B6 that inhibits AGE formation, showed moderate improvement in psychotic symptoms in patients with schizophrenia with high plasma pentosidine levels [7]. This evidence clearly indicates that AGEs play a key role in the development and pathophysiology of schizophrenia.

Cognitive impairment is one of the main clinical features of schizophrenia. To date, many studies have revealed a significant and stable association between schizophrenia and cognitive impairments, including working memory [8, 9], processing speed [9–11], and executive function [12]. These impairments have been observed even in first-episode psychosis (FEP) [13, 14]. Furthermore, cognitive impairment is related to poor functional outcomes [15–17], such as occupational prognosis, in schizophrenia [15, 18–20]. Importantly, therapeutic interventions targeting cognitive deficits have shown great success not only in the improvement of cognitive function [21, 22] but also in better functional outcomes [23], including occupational conditions [24–27]. In addition, cognitive remediation contributes to the improvement of negative symptoms [28], which is one of the main barriers to achieving recovery [29]. Therefore, it is essential that we understand the importance of cognitive impairment in order to promote the well-being of patients with schizophrenia.

To date, the relationship between AGEs and cognitive function in patients with schizophrenia is unknown. Thus, the aim of the present study was to identify the cognitive domain that is specifically affected by AGEs in order to facilitate functional recovery in patients with schizophrenia.

## Materials and methods

### Participants

Fifty-eight patients with schizophrenia (mean age: 46.8 ± 11.4 years; 41 men, 17 women), including three patients with schizoaffective disorder, were recruited from Matsuzawa Metropolitan Hospital (Setagaya-ku, Tokyo, Japan) and Takatsuki Hospital (Hachioji, Tokyo, Japan). Patients were diagnosed based on the criteria outlined in the Diagnostic and Statistical Manual of Mental Disorders, Fourth Edition. The cohort included 53 inpatients and five outpatients. The demographic and clinical characteristics of the patients are summarized in Table 1. All participants provided written informed consent, and the study protocols were approved by the ethics committees of both participating institutions (Tokyo Metropolitan Matsuzawa Hospital and Tokyo Metropolitan Institute of Medical Science; approval number 17–16). This study was conducted in accordance with the ethical principles of the Declaration of Helsinki.

**Table 1:** Participant characteristics.

Variables	*N*	Mean		SD
Age	58	46.8	±	11.4
Sex (male/female, n)	58	41	/	17
Body mass index (kg/m^2^)	55	23.1	±	4.1
Hemoglobin A1c (%)	56	5.3	±	0.4
eGFR (ml/min/1.73 m^2^)	57	79.9	±	14.6
Pentosidine (ng/ml)	58	72.8	±	50.4
Pyridoxal (ng/ml)	58	6.9	±	3.9
Inpatients/Outpatients (n)	58	53	/	5
Educational years (years)	58	12.4	±	2.9
Onset of disease (years old)	58	24.2	±	9.1
Disease duration (years)	58	22.6	±	12.3
Anti-psychotics (mg/day, CP equivalent)	58	846.3	±	648.2
MS-J (total)	58	4.6	±	4.7
MS-J (positive symptoms)	58	1.8	±	2.5
MS-J (negative symptoms)	58	0.9	±	1.5
MS-J (general symptoms)	58	1.9	±	2.0

Abbreviations. SD, Standard deviation; eGFR, estimated glomerular filtration rate; CP, Chlorpromazine; MS-J, Manchester Scale-Japanese version.

### Measurement of AGEs and other molecules

Fresh plasma and serum samples were collected from all participants. Plasma levels of pentosidine were determined using high-performance liquid chromatography (HPLC) as previously described [30]. In brief, plasma samples were lyophilized and hydrolyzed in 100 μL of 6 N hydrochloric acid for 16 h at 110°C under nitrogen. Then, the samples were neutralized with 100 μL of 5 N sodium hydroxide and 200 μL of 0.5 M sodium phosphate buffer (pH 7.4), filtered through a 0.5 μm filter, and finally diluted with phosphate-buffered saline. A sample, corresponding to 25 μg of protein, was injected into an HPLC system and fractionated on a C18 reverse-phase column. The effluent was monitored at excitation-emission wavelengths of 335/385 nm using a fluorescence detector (RF-10A; Shimadzu, Kyoto, Japan). Synthetic pentosidine was used as the reference standard to obtain a standard curve. Serum levels of the three forms of vitamin B6 (pyridoxine, pyridoxal, and pyridoxamine) were determined using HPLC at the private clinical laboratory test company SRL (Tokyo, Japan). Other parameters (glycohemoglobin A1C and creatinine) were measured in the blood samples. Glomerular filtration rate was estimated using the Modification of Diet in Renal Diseases study equation.

### Assessment of cognitive performance

The Wechsler Adult Intelligence Scale-Third Edition (WAIS-III), composed of verbal intelligence quotient (VIQ), performance IQ (PIQ), and full-scale IQ (FIQ), was used to assess the cognitive function of the patients. The WAIS-III measures four major index scores: (1) verbal comprehension, (2) perceptual organization, (3) working memory, and (4) processing speed.

The Wisconsin Card Sorting Test (WCST) was used to evaluate executive function. In the current study, we adopted the WCST-Keio-FS computer version (WCST-KFS). We selected five outcome measures in the WCST-KFS: (1) categories achieved (CA), (2) total errors (TE), (3) perseverative errors of Milner (PEM), (4) Perseverative errors of Nelson (PEN), and (5) difficulty of maintaining set (DMS).

### Assessment of psychotic symptoms

We used the Manchester Scale-Japanese version (MS-J), which was designed to assess the severity of schizophrenia symptoms [31]. It consists of eight sub-clusters, evaluated using a 5-point rating scale. Scores on four scales (depression, anxiety, hallucinations, and coherently expressed delusions) were determined based on patient responses, while scores on the remaining four scales (flattened affect, incongruous affect, psychomotor retardation, incoherence and irrelevance of speech, poverty of speech, and muteness) were determined via observation in the interview. All MS-J assessments were conducted by an experienced clinical psychologist.

### Clinical variables

Clinical information such as age, sex, body mass index, duration of illness, age of onset, duration of hospitalization, number of hospitalizations, level of education, and dose of antipsychotic medication were collected from a brief interview at the time of blood collection or from the clinical records of each patient.

### Statistical analysis

Demographic data are presented as the mean ± standard deviation (SD). Multiple regression analysis was used to examine whether AGEs were associated with processing speed. The significance level (α) was set to 0.05, for two-tailed tests. All statistical analyses were performed using the Statistical Package for the Social Sciences (version 20.0; IBM Corp., Armonk, NY, USA).

## Results

Table 1 shows the characteristics of the participants. Of the 58 patients, 53 (91%) were hospitalized. The mean disease duration and daily dose of antipsychotics were 24.2 (9.1) years (SD) and 846.3 (648.2) mg/day (SD, chlorpromazine equivalent), respectively. Correlation analysis revealed that only processing speed was significantly correlated with plasma pentosidine levels among the subscales of the WAIS-III (S1 Table, Spearman’s correlation coefficient = -0.35, *p* = 0.006), while no correlation was found between pentosidine and each item of WSCT (S1 Table). As shown in Table 2, multiple regression analysis adjusted for age, sex [32, 33], body mass index, educational years, daily dose of antipsychotics, and psychotic symptoms demonstrated that processing speed was significantly associated with plasma pentosidine levels (standardized β = -0.425, *p* = 0.009).

**Table 2:** Association between pentosidine and processing speed.

	Unadjusted model		Adjusted model 1^a^		Adjusted model 2^b^	
	Standardized β	*p*-value^c^	Standardized β	*p*-value^c^	Standardized β	*p*-value^c^
Pentosidine (ng/ml)	-0.379	0.009	-0.423	0.006	-0.425	0.009
Age (year-old)			0.188	0.465	0.186	0.594
Sex			-0.098	>.99	-0.109	>.99
Body mass index (%)			0.110	>.99	0.091	>.99
Educational years (years)			0.178	0.486	0.173	0.567
Anti-psychotics (mg/day, CP equivalent)					0.020	>.99
Manchester Scale (total score)					-0.048	>.99

Abbreviations: CP; Chlorpromazine.

^a^ Adjusted for age, sex, and educational years.

^b^ Adjusted for age, sex educational years, daily dose of anti-psychotics, and Manchester scale.

^c^ P values adjusted for multiple testing using the Bonferroni method.

## Discussion

In this study, we identified that processing speed is the key cognitive domain that is significantly affected by AGEs in patients with schizophrenia. The significance remains even after adjusting for the considerable confounding effects of age, sex, body mass index, educational history, psychological symptoms, and antipsychotic medications. To the best of our knowledge, this is the first study to specify the cognitive impairment associated with AGEs in patients with schizophrenia.

Our findings are consistent with previous reports indicating that processing speed impairment is related to schizophrenia [9–11] and FEP [13]. Furthermore, two longitudinal studies revealed that processing speed is linked to subsequent functional outcomes [16], including occupational status [20]. This evidence emphasizes that processing speed deficits are a potential intervention target to obtain better functional outcomes in patients with schizophrenia. Indeed, previous studies have demonstrated that cognitive intervention is effective for improving processing speed [22], even for inpatients with schizophrenia [34]. Taken together, these findings have important clinical implications. AGEs may be a valuable biological marker to specifically identify an impaired cognitive domain; therefore, anti-AGE therapy may be a useful treatment option to achieve functional recovery in patients with schizophrenia, especially those with increased AGEs.

The biological mechanism by which AGEs specifically impair processing speed remains to be clarified. First, we must discuss whether peripheral AGEs reflect brain AGEs; Akhter et al. reported that mice fed a high AGE diet showed higher AGE levels in both the serum and brain compared to mice fed a normal AGEs diet [35]. Furthermore, in a human post-mortem study, AGE levels in the cerebrospinal fluid correlated with plasma AGE levels [36]. These reports indicate that an increase in blood AGEs may reflect accumulation of brain AGEs. White matter abnormalities are one possible explanation for the association between AGEs and processing speed. Karbasforoushan et al. reported that processing speed impairment is mediated by white matter integrity [37]. Kochunov et al. showed that white matter disruption, measured by diffusion-weighted imaging, correlated with processing speed in patients with schizophrenia [38]. This relationship was also observed in siblings of patients and was not affected by antipsychotic medications [38]. Similarly, a relationship at the whole-brain level between white matter volume and processing speed was observed in drug-naïve, healthy young people [39]. Son et al. demonstrated a significant negative correlation between plasma pentosidine and white matter integrity in patients with schizophrenia [40]. Furthermore, a study using postmortem brains from patients with multiple sclerosis, white matter disease, showed that binding of AGEs to the receptor for AGEs (RAGE) triggers neuroinflammation and, consequently, leads to white matter damage [36]. The results of these studies suggest that neuroinflammation, which is induced by AGE-RAGE axis interaction, impairs processing speed via white matter disruption in schizophrenia.

Our findings suggest that AGE-reducing therapy may be effective in improving processing speed in patients with schizophrenia. Pyridoxamine, one of the three forms of vitamin B6, inhibits AGE formation by scavenging the AGE precursor. Our previous clinical study demonstrated that pyridoxamine improved psychotic symptoms in some patients with schizophrenia with high plasma pentosidine levels [7]. Furthermore, our data showed that there was a marginally positive association between serum pyridoxal levels and processing speed (S2 Table, Spearman’s correlation coefficient = 0.22, *p* = 0.093). These findings indicate the possibility that vitamin B6 administration is an effective approach for improving the processing speed in schizophrenia. Recent investigations have shown a significant association between AGEs and adverse lifestyle habits, including low physical activity [41] and high AGE diets [42]. In fact, aerobic exercise [43] and dietary intervention [44] improved cognitive function in patients with schizophrenia. Further studies are needed to examine the effect of anti-AGE therapies on processing speed and functional outcomes in patients with schizophrenia.

This study has several limitations. First, a cross-sectional design was unable to reveal the direction of the relationship between AGEs and cognitive impairment. A prospective design in future research is needed to address this issue. Second, we should consider the limited statistical power due to the relatively small sample size. However, the robust significance allows us to interpret that the association between AGEs and processing speed dysfunction is reliable.

## Conclusions

In conclusion, we found that the processing speed is specifically related to AGEs. We hope that interventions for AGEs, such as administration of vitamin B6 or modifications of adverse lifestyles, will be useful treatment options to improve patient recovery and cognitive function.

## Supporting information

S1 Table Correlation between pentosidine and cognitive function. (DOCX)

S2 Table Correlation between pyridoxal and cognitive performance. (DOCX)

S1 Raw data (XLSX)

## References

[1] Hanssen NM, Beulens JW, van Dieren S, Scheijen JL, van der AD, Spijkerman AM et al. Plasma advanced glycation end products are associated with incident cardiovascular events in individuals with type 2 diabetes: a case-cohort study with a median follow-up of 10 years (EPIC-NL). Diabetes. 2015;64: 257–265. 10.2337/db13-1864 24848072

[2] Miyata T, van Ypersele de Strihou C, Kurokawa K, Baynes JW. Alterations in nonenzymatic biochemistry in uremia: origin and significance of "carbonyl stress" in long-term uremic complications. Kidney Int. 1999;55: 389–399. 10.1046/j.1523-1755.1999.00302.x 9987064

[3] Meli M, Perier C, Ferron C, Parssegny F, Denis C, Gonthier R et al. Serum pentosidine as an indicator of Alzheimer’s disease. J Alzheimers Dis. 2002;4: 93–96. 10.3233/jad-2002-4203 12214132

[4] Arai M, Yuzawa H, Nohara I, Ohnishi T, Obata N, Iwayama Y et al. Enhanced carbonyl stress in a subpopulation of schizophrenia. Arch Gen Psychiatry. 2010;67: 589–597. 10.1001/archgenpsychiatry.2010.62 20530008

[5] Miyashita M, Arai M, Yuzawa H, Niizato K, Oshima K, Kushima I et al. Replication of enhanced carbonyl stress in a subpopulation of schizophrenia. Psychiatry Clin Neurosci. 2014;68: 83–84. 10.1111/pcn.12081 24393354

[6] Miyashita M, Arai M, Kobori A, Ichikawa T, Toriumi K, Niizato K et al. Clinical features of schizophrenia with enhanced carbonyl stress. Schizophr Bull. 2014;40: 1040–1046. 10.1093/schbul/sbt129 24062594 PMC4133661

[7] Itokawa M, Miyashita M, Arai M, Dan T, Takahashi K, Tokunaga T et al. Pyridoxamine: A novel treatment for schizophrenia with enhanced carbonyl stress. Psychiatry Clin Neurosci. 2018;72: 35–44. 10.1111/pcn.12613 29064136

[8] Forbes NF, Carrick LA, McIntosh AM, Lawrie SM. Working memory in schizophrenia: a meta-analysis. Psychol Med. 2009;39: 889–905. 10.1017/S0033291708004558 18945379

[9] Michel NM, Goldberg JO, Heinrichs RW, Miles AA, Ammari N, McDermid Vaz S. WAIS-IV profile of cognition in schizophrenia. Assessment. 2013;20: 462–473. 10.1177/1073191113478153 23443820

[10] Dickinson D, Ramsey ME, Gold JM. Overlooking the obvious: a meta-analytic comparison of digit symbol coding tasks and other cognitive measures in schizophrenia. Arch Gen Psychiatry. 2007;64: 532–542. 10.1001/archpsyc.64.5.532 17485605

[11] Knowles EE, David AS, Reichenberg A. Processing speed deficits in schizophrenia: reexamining the evidence. Am J Psychiatry. 2010;167: 828–835. 10.1176/appi.ajp.2010.09070937 20439390

[12] Thai ML, Andreassen AK, Bliksted V. A meta-analysis of executive dysfunction in patients with schizophrenia: Different degree of impairment in the ecological subdomains of the Behavioural Assessment of the Dysexecutive Syndrome. Psychiatry Res. 2019;272: 230–236. 10.1016/j.psychres.2018.12.088 30590277

[13] Fatouros-Bergman H, Cervenka S, Flyckt L, Edman G, Farde L. Meta-analysis of cognitive performance in drug-naïve patients with schizophrenia. Schizophr Res. 2014;158: 156–62. 10.1016/j.schres.2014.06.034 25086658

[14] Hwang WJ, Lee TY, Shin WG, Kim M, Kim J, Lee J et al. Global and Specific Profiles of Executive Functioning in Prodromal and Early Psychosis. Front Psychiatry. 2019;10: 356. 10.3389/fpsyt.2019.00356 31178768 PMC6537881

[15] Green MF, Kern RS, Braff DL, Mintz J. Neurocognitive deficits and functional outcome in schizophrenia: are we measuring the "right stuff"? Schizophr Bull Open. 2000;26: 119–136. 10.1093/oxfordjournals.schbul.a033430 10755673

[16] Milev P, Ho BC, Arndt S, Andreasen NC. Predictive values of neurocognition and negative symptoms on functional outcome in schizophrenia: a longitudinal first-episode study with 7-year follow-up. Am J Psychiatry. 2005;162: 495–506. 10.1176/appi.ajp.162.3.495 15741466

[17] Green MF. Impact of cognitive and social cognitive impairment on functional outcomes in patients with schizophrenia. J Clin Psychiatry. 2016;77: 8–11. 10.4088/JCP.14074su1c.02 26919052

[18] McGurk SR, Mueser KT, Harvey PD, LaPuglia R, Marder J. Cognitive and symptom predictors of work outcomes for clients with schizophrenia in supported employment. Psychiatr Serv. 2003;54: 1129–1135. 10.1176/appi.ps.54.8.1129 12883141

[19] McGurk SR, Mueser KT. Cognitive and clinical predictors of work outcomes in clients with schizophrenia receiving supported employment services: 4-year follow-up. Adm Policy Ment Health. 2006;33: 598–606. 10.1007/s10488-006-0070-2 16799831

[20] Pothier W, Cellard C, Corbière M, Villotti P, Achim AM, Lavoie A et al. Determinants of occupational outcome in recent-onset psychosis: The role of cognition. Schizophr Res Cogn. 2019;18: 100158. 10.1016/j.scog.2019.100158 31463205 PMC6710235

[21] Hogarty GE, Flesher S, Ulrich R, Carter M, Greenwald D, Pogue-Geile M et al. Cognitive enhancement therapy for schizophrenia: effects of a 2-year randomized trial on cognition and behavior. Arch Gen Psychiatry. 2004;61: 866–876. 10.1001/archpsyc.61.9.866 15351765

[22] McGurk SR, Twamley EW, Sitzer DI, McHugo GJ, Mueser KT. A meta-analysis of cognitive remediation in schizophrenia. Am J Psychiatry. 2007;164: 1791–1802. 10.1176/appi.ajp.2007.07060906 18056233 PMC3634703

[23] Wykes T, Huddy V, Cellard C, McGurk SR, Czobor P. A meta-analysis of cognitive remediation for schizophrenia: methodology and effect sizes. Am J Psychiatry. 2011;168: 472–485. 10.1176/appi.ajp.2010.10060855 21406461

[24] Wexler BE, Bell MD. Cognitive remediation and vocational rehabilitation for schizophrenia. Schizophr Bull Open. 2005;31: 931–941. 10.1093/schbul/sbi038 16079390

[25] McGurk SR, Mueser KT, DeRosa TJ, Wolfe R. Work, recovery, and comorbidity in schizophrenia: a randomized controlled trial of cognitive remediation. Schizophr Bull Open. 2009;35: 319–335. 10.1093/schbul/sbn182 PMC2659315 19269925

[26] McGurk SR, Mueser KT, Xie H, Welsh J, Kaiser S, Drake RE et al. Cognitive Enhancement Treatment for People With Mental Illness Who Do Not Respond to Supported Employment: A Randomized Controlled Trial. Am J Psychiatry. 2015;172: 852–861. 10.1176/appi.ajp.2015.14030374 25998278

[27] Ikebuchi E, Sato S, Yamaguchi S, Shimodaira M, Taneda A, Hatsuse N et al. Does improvement of cognitive functioning by cognitive remediation therapy effect work outcomes in severe mental illness? A secondary analysis of a randomized controlled trial. Psychiatry Clin Neurosci. 2017;71: 301–308. 10.1111/pcn.12486 27873453

[28] Cella M, Preti A, Edwards C, Dow T, Wykes T. Cognitive remediation for negative symptoms of schizophrenia: A network meta-analysis. Clin Psychol Rev. 2017;52: 43–51. 10.1016/j.cpr.2016.11.009 27930934

[29] Van Eck RM, Burger TJ, Vellinga A, Schirmbeck F, de Haan L. The Relationship Between Clinical and Personal Recovery in Patients With Schizophrenia Spectrum Disorders: A Systematic Review and Meta-analysis. Schizophr Bull. 2018;44: 631–642. 10.1093/schbul/sbx088 29036720 PMC5890469

[30] Miyata T, Taneda S, Kawai R, Ueda Y, Horiuchi S, Hara M et al. Identification of pentosidine as a native structure for advanced glycation end products in beta-2-microglobulin-containing amyloid fibrils in patients with dialysis-related amyloidosis. Proc Natl Acad Sci U S A. 1996;93: 2353–2358. 10.1073/pnas.93.6.2353 8637877 PMC39800

[31] Hyde CE. The Manchester Scale. A standardised psychiatric assessment for rating chronic psychotic patients. Br J Psychiatry Suppl. 1989: 45–48. 2619981

[32] Huang X, Bao C, Lv Q, Zhao J, Wang Y, Lang X et al. Sex difference in cognitive impairment in drug-free schizophrenia: Association with miR-195 levels. Psychoneuroendocrinology. 2020;119: 104748. 10.1016/j.psyneuen.2020.104748 32559610

[33] Mu L, Liang J, Wang H, Chen D, Xiu M, Zhang XY. Sex differences in association between clinical correlates and cognitive impairment in patients with chronic schizophrenia. J Psychiatr Res. 2020;131: 194–202. 10.1016/j.jpsychires.2020.09.003 32980647

[34] Cella M, Price T, Corboy H, Onwumere J, Shergill S, Preti A. Cognitive remediation for inpatients with psychosis: a systematic review and meta-analysis. Psychol Med. 2020;50: 1062–1076. 10.1017/S0033291720000872 32349802

[35] Akhter F, Chen D, Akhter A, Sosunov AA, Chen A, McKhann GM et al. High Dietary Advanced Glycation End Products Impair Mitochondrial and Cognitive Function. J Alzheimers Dis. 2020;76: 165–178. 10.3233/JAD-191236 32444539 PMC7581068

[36] Wetzels S, Vanmierlo T, Scheijen J, van Horssen J, Amor S, Somers V et al. Methylglyoxal-Derived Advanced Glycation Endproducts Accumulate in Multiple Sclerosis Lesions. Front Immunol. 2019;10: 855. 10.3389/fimmu.2019.00855 31068938 PMC6491451

[37] Karbasforoushan H, Duffy B, Blackford JU, Woodward ND. Processing speed impairment in schizophrenia is mediated by white matter integrity. Psychol Med. 2015;45: 109–20. 10.1017/S0033291714001111 25066842 PMC5297385

[38] Kochunov P, Rowland LM, Fieremans E, Veraart J, Jahanshad N, Eskandar G et al. Diffusion-weighted imaging uncovers likely sources of processing-speed deficits in schizophrenia. Proc Natl Acad Sci U S A. 2016;113: 13504–13509. 10.1073/pnas.1608246113 27834215 PMC5127361

[39] Magistro D, Takeuchi H, Nejad KK, Taki Y, Sekiguchi A, Nouchi R et al. The Relationship between Processing Speed and Regional White Matter Volume in Healthy Young People. PloS One. 2015;10: e0136386. 10.1371/journal.pone.0136386 26397946 PMC4580478

[40] Son S, Arai M, Miyata J, Toriumi K, Mizuta H, Hayashi T et al. Enhanced carbonyl stress and disrupted white matter integrity in schizophrenia. Schizophr Res. 2020;223: 242–248. 10.1016/j.schres.2020.08.007 32843203

[41] Isami F, West BJ, Nakajima S, Yamagishi SI. Association of advanced glycation end products, evaluated by skin autofluorescence, with lifestyle habits in a general Japanese population. J Int Med Res. 2018;46: 1043–1051. 10.1177/0300060517736914 29322837 PMC5972252

[42] Uribarri J, Cai W, Woodward M, Tripp E, Goldberg L, Pyzik R et al. Elevated serum advanced glycation endproducts in obese indicate risk for the metabolic syndrome: a link between healthy and unhealthy obesity? J Clin Endocrinol Metab. 2015;100: 1957–1966. 10.1210/jc.2014-3925 25695886 PMC4422896

[43] Firth J, Stubbs B, Rosenbaum S, Vancampfort D, Malchow B, Schuch F et al. Aerobic Exercise Improves Cognitive Functioning in People With Schizophrenia: A Systematic Review and Meta-Analysis. Schizophr Bull Open. 2017;43: 546–556. 10.1093/schbul/sbw115 27521348 PMC5464163

[44] Adamowicz K, Mazur A, Mak M, Samochowiec J, Kucharska-Mazur J. Metabolic Syndrome and Cognitive Functions in Schizophrenia-Implementation of Dietary Intervention. Front Psychiatry. 2020;11: 359. 10.3389/fpsyt.2020.00359 32425834 PMC7203414

