# Supplementary material for: Advanced glycation end products and cognitive impairment in schizophrenia
Source: PLoS One. 2021 May 26;16(5):e0251283. doi: 10.1371/journal.pone.0251283 (PMC8153415; doi:10.1371/journal.pone.0251283)
Supplement: S1 Table — (DOCX) [file pone.0251283.s001.docx]

S1 Table.

| **S1 Table. Correlation between pentosidine and cognitive function** | | | |
| --- | --- | --- | --- |
| Cognitive function | | Correlation coefficient | *p*-value |
| Wechsler Adult Intelligence Scale III | |  |  |
|  | Verbal comprehension | -0.17 | 0.202 |
|  | Working memory | -0.08 | 0.546 |
|  | Perceptual organization | -0.18 | 0.184 |
|  | Processing speed* | -0.35 | 0.006 |
| Wisconsin Card Sorting Test | |  |  |
|  | Categories achieved | -0.15 | 0.261 |
|  | Total errors | 0.09 | 0.492 |
|  | Perseverative errors of Milner | 0.23 | 0.079 |
|  | Perseverative errors of Nelson | 0.19 | 0.163 |
|  | Difficulties of maintaining set | 0.16 | 0.226 |
| Abbreviations. IQ, Intelligence quotient.  * *p* <.05 (Bonferroni correction) | | | |
