# Supplementary material for: Advanced glycation end products and cognitive impairment in schizophrenia
Source: PLoS One. 2021 May 26;16(5):e0251283. doi: 10.1371/journal.pone.0251283 (PMC8153415; doi:10.1371/journal.pone.0251283)
Supplement: S2 Table — (DOCX) [file pone.0251283.s002.docx]

S2 Table.

| **S2 Table. Correlation between pyridoxal and cognitive performance** | | |
| --- | --- | --- |
|  | Correlation coefficient | *p*-value |
| Verbal comprehension | 0.105 | 0.431 |
| Perceptual organization | 0.176 | 0.186 |
| Working memory | 0.191 | 0.151 |
| Processing speed | 0.223 | 0.093 |
| Abbreviations. IQ, Intelligence quotient. | | |
